# Biomechanical and Viscoelastic Properties of the Ankle Muscles in Relation to Muscle Force in Patients with Operated Tibial Pilon Fractures

**DOI:** 10.3390/jcm15082934

**Published:** 2026-04-12

**Authors:** Andrei-Daniel Bolovan, Roxana-Ramona Onofrei, Gheorghe-Bogdan Hogea, Ahmed Abu-Awwad, Jenel-Marian Patrascu, Alexandra-Roxana Tapardea, Alexandru-Florian Crisan, Elena-Constanta Amaricai

**Affiliations:** 1 Doctoral School, “Victor Babes” University of Medicine and Pharmacy Timisoara, 300041 Timisoara, Romania; andrei.bolovan@umft.ro (A.-D.B.); roxana.tapardea@umft.ro (A.-R.T.); 2 Research Center for Assessment of Human Motion, Functionality and Disability, Department of Rehabilitation, Physical Medicine and Rheumatology, “Victor Babes” University of Medicine and Pharmacy Timisoara, 300041 Timisoara, Romania; onofrei.roxana@umft.ro (R.-R.O.); crisan@umft.ro (A.-F.C.); amaricai.elena@umft.ro (E.-C.A.); 3 Department of Orthopedics and Traumatology, “Victor Babes” University of Medicine and Pharmacy Timisoara, 300041 Timisoara, Romania; ahm.abuawwad@umft.ro (A.A.-A.); jenel.patrascu@umft.ro (J.-M.P.J.); 4 “Pius Brinzeu” Emergency Clinical County Hospital, Bld Liviu Rebreanu, No. 156, 300723 Timisoara, Romania; 5 Research Center Teodor Sora, Department of Orthopedics II, “Victor Babes” University of Medicine and Pharmacy Timisoara, Eftimie Murgu Square, No. 2, 300041 Timisoara, Romania; 6 Pulmonary Rehabilitation Center, Clinical Hospital of Infectious Diseases and Pulmonology, “Victor Babes”, 300310 Timisoara, Romania

**Keywords:** tibial pilon fracture, muscle stiffness, viscoelastic muscle properties, stress relaxation time, isometric muscle strength, ankle biomechanics, muscle–force relationship

## Abstract

**Background:** Tibial pilon fractures are complex injuries frequently associated with persistent functional impairment, even after successful surgical fixation. While previous studies have reported deficits in muscle strength and balance, little is known about the side-to-side variations in intrinsic biomechanical and viscoelastic muscle properties following surgery. **Objectives**: This study aimed to compare the biomechanical and viscoelastic properties of ankle periarticular muscles between the affected and non-affected limbs in patients with surgically treated unilateral tibial pilon fractures. A secondary objective was to evaluate the relationship between intrinsic muscle properties and isometric muscle force. **Methods:** A total of 39 subjects with unilateral surgically treated tibial pilon fractures were evaluated after fracture healing. Myotonometric assessment was performed to evaluate muscle mechanical parameters, including tone (frequency), stiffness, and elasticity (decrement), as well as viscoelastic properties, including relaxation time and creep, in the tibialis anterior, peroneus longus, medial gastrocnemius, and lateral gastrocnemius muscles. Isometric muscle force of ankle dorsiflexors and plantar flexors was measured using a handheld dynamometer. Side-to-side comparisons and Pearson correlation analyses were performed. **Results:** The affected limb showed significantly reduced ankle range of motion in all planes and significantly lower isometric muscle force in both the dorsiflexors (*p* = 0.0002) and the plantar flexors (*p* = 0.0066). Stiffness was significantly higher in the medial (*p* = 0.038) and lateral gastrocnemius (*p* = 0.045) muscles on the affected side. Decrement was significantly increased (indicating reduced elasticity) in the peroneus longus (*p* = 0.021). No significant differences were observed for tone, relaxation time, or creep. **Conclusions:** Myotonometry revealed increased stiffness in the gastrocnemius muscles and reduced elasticity in the peroneus longus on the operated side compared with the non-affected limb. Tone and viscoelastic properties did not differ significantly between sides. However, tone, stiffness, and elasticity were significantly correlated with muscle force, indicating a relationship between intrinsic muscle mechanical properties and force production after tibial pilon fracture surgery.

## 1. Introduction

Tibial pilon fractures involve the distal tibia and are most commonly the result of high-energy mechanisms, such as falls from height or motor vehicle accidents. Because the injury can affect both the joint surface and the surrounding soft tissues, treatment is challenging and frequently associated with prolonged recovery. Even when anatomical reduction and stable fixation are achieved, many patients continue to report impairments in ankle mobility and overall functional performance during long-term follow-up [1,2].

Most of the research on tibial pilon fractures has primarily focused on fracture management and surgical techniques, fracture classification, and radiological outcomes [3,4,5]; however, the intrinsic biomechanical and viscoelastic properties of the periarticular muscles of the ankle following surgery remain poorly understood. This highlights a significant gap in the literature because soft-tissue adaptations might persist after fracture healing and could affect functional outcomes [6].

It is increasingly recognized that functional recovery depends not only on bone healing but also on neuromuscular factors. Therefore, recent research has begun to explore aspects such as muscle strength, proprioception, and postural control after injury [7,8]. Previous work from our research group demonstrated that individuals treated surgically for tibial pilon fractures may present persistent reductions in ankle muscle strength as well as impairments in postural stability when compared with healthy individuals. These findings emphasize the significance of evaluating muscular function as part of the long-term assessment of patients recovering from this injury [9,10].

Reduced ankle isometric muscle strength following an injury weakens the muscles’ capacity to ensure joint stability during weight-bearing tasks, while associated proprioceptive deficits may further impair postural control and dynamic ankle stability [11]. In addition, impaired ankle muscle function may alter neuromuscular control strategies during demanding tasks such as landing, reducing the ability to attenuate impact forces effectively. A review by Lin et al. highlighted that individuals with ankle instability exhibit altered neuromuscular activation and changes in ground reaction force patterns during landing, indicating reduced dynamic control of the ankle joint [12].

Muscle function depends not only on neural activation and contractile capacity, but also on the intrinsic biomechanical and viscoelastic properties of muscle tissue. Parameters such as muscle stiffness, elasticity or viscoelasticity influence how forces are generated and transmitted through joints. Consequently, changes in these properties can impair functional recovery after trauma [13,14].

After traumatic injury and surgery, periarticular muscles may undergo structural adaptations due to immobilization, altered loading patterns, and inflammatory processes. These factors may promote changes in the extracellular matrix, including collagen remodeling and changes in fascial stiffness. Such structural changes may influence both the passive mechanical behavior of muscle tissue and its ability to generate force during voluntary contraction [15,16].

Myotonometric assessment is a non-invasive method that facilitates the assessment of several mechanical characteristics of muscle tissue at the resting state, including muscle tone, stiffness, elasticity, relaxation time, and creep. These parameters provide information about the biomechanical state of muscle tissue and can help identify long-term adaptations that occur after injury or surgical treatment [17,18]. Therefore, combining myotonometric assessment with muscle strength evaluation may provide a more comprehensive understanding of muscle function after tibial pilon fracture surgery.

The review by McGowen et al. [17] on the utility of myotonometry in musculoskeletal rehabilitation indicates that when the objective is to improve muscle function rather than to achieve high force production (as in the initial stages of postoperative period rehabilitation), myotonometry might be a useful instrument. This instrument can enhance a clinician’s capacity to diagnose muscle dysfunction and to monitor the efficacy of interventions.

The shear wave elastography facilitates a real-time visualization of muscle stiffness during active or passive muscle movement [19]. This investigation is expensive, requires more extensive operator training, and is not easily portable. Instead, the MyotonPRO is a novel handheld device developed as a portable method for assessing muscle mechanical properties as a simpler alternative to shear wave elastography [20].

The main purpose of this study was to explore the biomechanical and viscoelastic properties of the ankle muscles in patients after unilateral tibial pilon fracture surgery, comparing the affected and non-affected sides. Another objective was to assess correlations between ankle muscle properties (muscle force and myotonometric parameters). We hypothesized that patients who had suffered a surgically treated tibial pilon fracture would exhibit higher tone and stiffness and lower elasticity in the ankle periarticular muscles of the injured lower limb compared with the healthy lower limb. To our knowledge, no previous study has specifically investigated side-to-side differences in ankle muscle myotonometric properties and their relationship with isometric muscle force in this population.

## 2. Materials and Methods

### 2.1. Study Design

**Figure 1:**
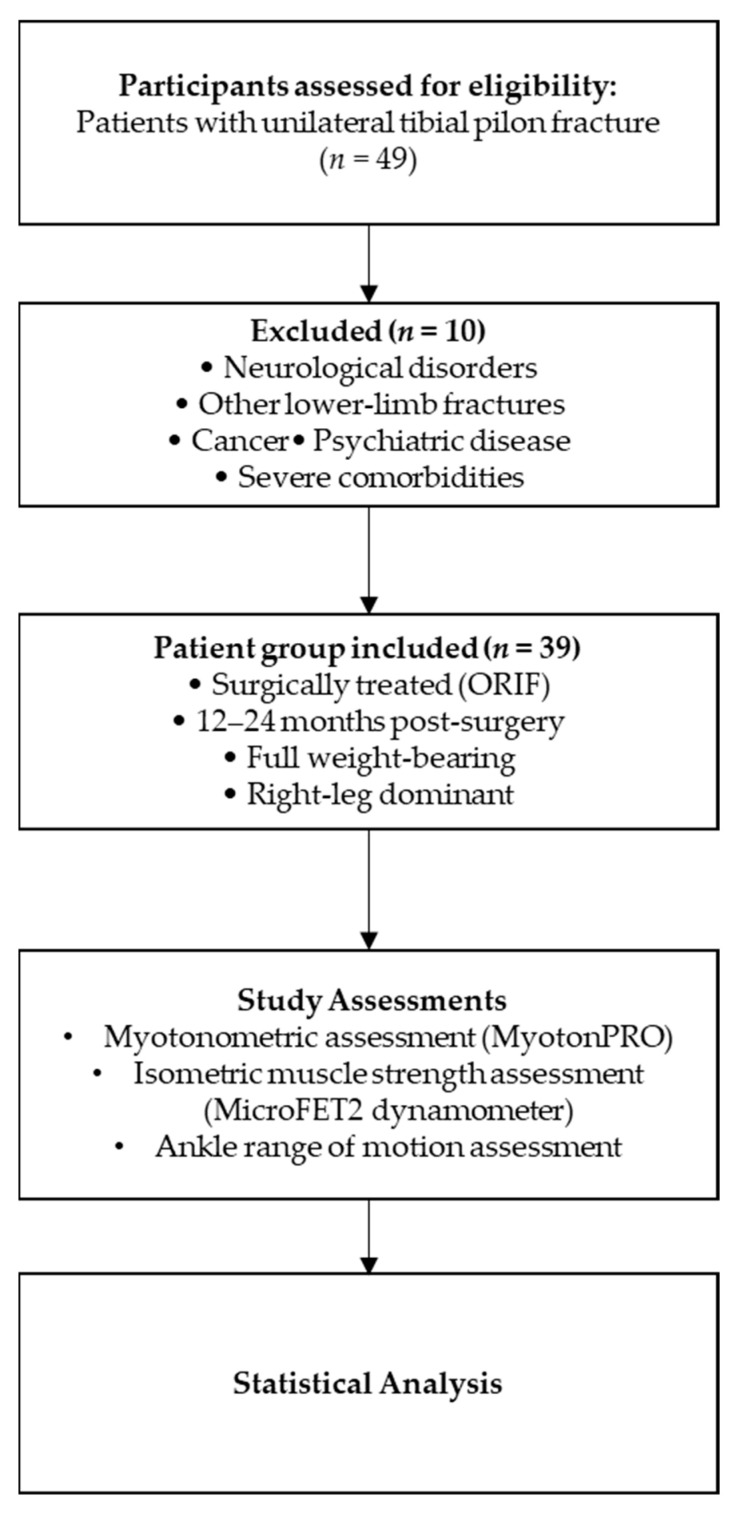
Flow diagram of the study design and assessment protocol.

A cross-sectional paired analysis was conducted to examine differences between the affected and non-affected sides in patients who previously sustained a unilateral tibial pilon fracture treated surgically. The study protocol included clinical evaluation, myotonometric assessment of muscle biomechanical and viscoelastic properties, and measurement of isometric muscle strength (Figure 1).

### 2.2. Participants

A total of 49 patients who received surgical treatment for unilateral tibial pilon fractures were initially screened for eligibility. Assessments were performed between 12 and 24 months after surgery, facilitating complete fracture consolidation and functional recovery. Participants were included if radiological healing was confirmed and full weight-bearing on the affected limb had been achieved.

Exclusion criteria consisted of previous trauma or fractures to either lower limb, neurological disorders or other health conditions affecting gait or muscle function, and leg length discrepancy unrelated to the tibial pilon fracture. Patients with health conditions that could interfere with compliance and follow-up were also excluded (e.g., psychiatric disorders, cancer, severe cardiovascular problems, or morbid obesity). Ten patients were excluded due to specific conditions: lumbar disk hernia (*n* = 3), stroke (*n* = 2), hip fracture (*n* = 1), contralateral ankle fracture (*n* = 1), calcaneus fracture (*n* = 1), cancer (*n* = 1), and depression (*n* = 1).

Sample size calculation was performed for an effect size of 0.5, a significance level α = 0.05, and the statistical power of 0.8. Calculated by G*Power 3.1.9.7 software (Universität Kiel, Kiel, Germany), the needed sample size was of minimum 35 patients. The patients’ data are presented in Table 1. The distribution of injured sides revealed that 24 individuals had the fracture on their right side, while 15 had it on their left side.

When comparing ankle range of motion, we found that on the affected side, all movements were significantly reduced compared to the non-affected ankle (Table 2).

All subjects in the study underwent open reduction and internal fixation (ORIF). When there was significant soft-tissue injury or swelling, a temporary external fixation was initially applied, followed by definitive ORIF once soft-tissue conditions allowed. Fixation was carried out using anterior or anterolateral locking plates and screws under fluoroscopic guidance. Participation was voluntary, and all participants gave written informed consent. The study received approval from the Ethics Committee of “Victor Babes” University of Medicine and Pharmacy in Timisoara, Romania (reference no. 26/2023-08-25) and adhered to the Helsinki Declaration. Clinical data, including age, height, weight, and body mass index, were documented.

### 2.3. Assessment

The biomechanical and viscoelastic characteristics of the ankle muscles were evaluated using the MyotonPRO digital palpation device with software version 5.0.0.232 [18]. Measurements targeted the main ankle muscles, from each compartment: tibialis anterior (anterior compartment), the gastrocnemius medial and lateral (posterior compartment), and the peroneus longus (lateral compartment).

Participants lay supine for tibialis anterior and peroneus longus assessments, and prone for gastrocnemius evaluation. All the assessments were conducted in a relaxed state. The probe was placed perpendicular to the skin at the thickest part of each muscle belly, using standardized anatomical landmarks for consistent measurements [21,22]. For the tibialis anterior, the measurement point was at the proximal third of the distance from the tibial tuberosity to the lateral malleolus. For the peroneus longus, it was at the proximal third between the fibular head and the fibular malleolus. For the gastrocnemius (medial and lateral heads), measurements were taken at the midpoint between the popliteal fossa and the calcaneal insertion. The device applied brief mechanical impulses to determine tone, stiffness, elasticity (decrement), relaxation time, and creep [22].

Utilizing the handheld MicroFET2 dynamometer manufactured by Hoggan Scientific, a portable, accurate device that provides objective, reliable, and quantifiable muscle-testing results [23,24,25], we measured isometric muscle force. The device measures force in newtons (N), which represents the isometric force produced during muscle contraction. Participants, barefoot and lying supine with hips and knees extended and ankles neutral, performed ankle plantarflexion and dorsiflexion. The dynamometer was positioned on the sole over the metatarsal heads for plantar flexion and on the top of the foot for dorsiflexion with the examiner applying steady resistance during 3 s contractions. [24,25,26]. Each test was repeated three times, with 5 s of rest between trials. The average of these three measures was used for analysis. Muscle strength was assessed for both the affected and unaffected lower limbs.

### 2.4. Statistical Analysis

All statistical analyses were conducted with GraphPad Prism version 10.6.1 [27]. Descriptive statistics, including mean and standard deviation, were calculated for all variables. Prior to analysis, the normality of the data was checked with the D’Agostino-Pearson test. Differences in myotonometric parameters and isometric muscle force between affected and unaffected sides were assessed using Student’s unpaired *t*-test, with Welch’s correction. The relationship between myotonometric parameters (tone, stiffness, decrement) and isometric muscle force was analyzed with Pearson‘s rank correlation coefficient. A *p*-value less than 0.05 was considered statistically significant [28].

## 3. Results

We compared the myotonometric parameters of the ankle muscles between the affected and non-affected side (Table 3). The analysis revealed that the anterior tibialis was the only muscle for which no significant differences in the biomechanical properties (stiffness and elasticity) were found between the injured part and the healthy one (*p* > 0.05). Both the medial and lateral gastrocnemius muscles exhibited significantly increased stiffness, but not elasticity. In contrast, the longus peroneus evidenced a significantly higher decrement (thus a lower elasticity), with no significant difference in stiffness. Regarding the tone (state of tension) and viscoelastic properties (stress relaxation time and creep), no significant differences were noted for anterior tibialis, longus peroneus, medial, and lateral gastrocnemius muscles between the affected and non-affected side.

When comparing the isometric muscle force of the dorsiflexors and plantar flexors of the injured side and healthy one, we recorded statistically significant differences, with lower values for both the dorsiflexors and plantar flexors of the affected lower limb (Table 4).

We performed correlations between the muscle properties and isometric muscle force. We correlated the muscle tone, stiffness, and decrement (properties assessed in the relaxed state) with isometric muscle force (isometric voluntary contraction). We correlated the anterior tibialis properties with the isometric muscle force of dorsiflexors, and the medial and lateral gastrocnemius properties with the isometric muscle force of plantar flexors. Correlations were performed for the muscles on both the affected and non-affected sides (Table 5).

For the injured lower limb, the results showed that the isometric muscle force of the dorsiflexors was significantly positively correlated with the anterior tibialis muscle’s tone (*p* = 0.003) and stiffness (*p* = 0.0001). On the healthy side, we noted that the isometric muscle force of the dorsiflexors was significantly positively correlated with the tone and stiffness of the anterior tibialis muscle (tone: *p* < 0.0001; stiffness: *p* < 0.0001). In addition to that, for the non-affected side, the isometric muscle force of plantar flexors was significantly positively correlated with tone and stiffness, respectively, of the medial gastrocnemius muscle (tone: *p* = 0.0018; stiffness: *p* = 0.003), and with the stiffness of the lateral gastrocnemius muscle (*p* = 0.01). However, the isometric muscle force of plantar flexors was significantly negatively correlated with the decrement of the lateral gastrocnemius muscle of the affected side (*p* = 0.017) and the non-affected side, respectively (*p* = 0.002) (Table 5).

Although not statistically significant, all the correlations of tone and stiffness were positive for the anterior tibialis, medial, and lateral gastrocnemius muscles. The higher the tone and stiffness of the muscle in a relaxed state, the greater the isometric muscle force of the muscle group that performs the same movement. In contrast, for the decrement, all the correlations were inverse. The lower the decrement (thus the higher the elasticity) in a relaxed state, the higher the isometric muscle force.

## 4. Discussion

This study aims to assess the impact of a unilateral tibial pilon fracture treated surgically on the biomechanical and viscoelastic properties of the ankle periarticular muscles, namely the anterior tibialis, longus peroneus, medial gastrocnemius, and lateral gastrocnemius. We compared intrinsic muscle properties of the same muscle group between the affected and non-affected sides.

Our results show a lower tone (intrinsic muscle tension at rest) in all four assessed muscles of the injured lower limb compared with healthy ones. The mean differences between the affected and non-affected sides (anterior tibialis: 0.56 Hz; longus peroneus: 0.27 Hz; medial gastrocnemius: 0.72 Hz; lateral gastrocnemius: 1.09 Hz) were closely similar with those recorded in the study of Stefaniak W et al. [8] (anterior tibialis: 1.6 Hz; longus peroneus: 1.8 Hz; medial gastrocnemius: 0.9 Hz; lateral gastrocnemius: 1.1 Hz). They compared the mechanical properties of the above-mentioned muscles between male athletes with chronic ankle instability and healthy, age-matched male athletes. The presence of fixation hardware following ORIF should also be considered when interpreting these findings. Although myotonometric measurements were performed at standardized muscle-belly sites, potential indirect effects of postoperative soft-tissue changes cannot be excluded.

Considering the stiffness, our data evidenced that both medial and lateral gastrocnemius muscles were significantly stiffer for the injured part in comparison to the healthy one. For anterior tibialis and longus peroneus, the stiffness was also higher on the affected side, but not statistically significant.

The increased stiffness observed in the gastrocnemius muscles of the operated limb might be explained by the mechanisms of connective tissue remodeling. The study by Langevin et al. [29] proposed an integrative pathophysiological model in which changes in mechanical loading, reduced mobility, and inflammation lead to adaptive remodeling of connective tissues, characterized by increased collagen deposition and fibrosis. These structural changes can increase tissue stiffness and disrupt the normal biomechanical function of the muscle-fascia complex. In patients with tibial pilon fractures, prolonged immobilization, postoperative inflammation, and altered neuromuscular activation patterns during recovery may create a biological environment that promotes similar connective tissue changes in the periarticular muscles of the ankle.

Another key factor potentially influencing these adaptations is the changed mechanical environment of the ankle joint following trauma. Prior research on chronic ankle instability indicates that repeated mechanical stress and accumulated microtrauma can lead to structural changes in both articular and periarticular tissues [30].

This interpretation is consistent with our findings, which showed significantly higher stiffness in the medial and lateral gastrocnemius muscles on the affected side. In addition, the positive correlation between muscle stiffness and isometric muscle strength suggests that these structural changes may also affect the periarticular muscles’ ability to produce force.

The study of Zügel M et al. [31] suggests that force can be transmitted through intermuscular and extramuscular fascial connections, forming a continuous mechanical network within the musculoskeletal system. The effectiveness of this force transmission depends partly on the mechanical characteristics of myofascial tissues, such as their stiffness and extracellular matrix organization. Changes in these properties, such as heightened stiffness or fibrosis after trauma or surgery, can alter how forces are distributed and their strength between muscles and adjacent tissues.

In our study, the decrement was higher for all the tested periarticular ankle muscles of the injured lower limb in comparison to the healthy one. The decrement of tissue natural oscillation inversely describes elasticity. Therefore, the elasticity of all four muscles was lower for the affected side.

Reduced elasticity of periarticular muscles following trauma or surgical intervention may also be explained by structural changes occurring in the surrounding fascial and connective tissues. After tissue injury, inflammatory processes stimulate fibroblast activity and increased collagen deposition within the extracellular matrix, leading to fibrosis and scar tissue formation. These fibrotic changes modify the mechanical properties of the affected tissues, reducing tissue compliance [15,16,31].

The mechanical stress relaxation time characterizes tissue recovery from displacement. The higher the tissue tension, the faster the tissue recovers its shape (a lower stress relaxation time) [32]. When compared to the healthy lower limb, the stress relaxation time of anterior tibialis, longus peroneus, medial, and lateral gastrocnemius was higher for the affected lower limb. This is in line with the lower tone (intrinsic tension) of the ankle periarticular muscles on the injured part.

In our study, we also analyzed correlations between the isometric muscle force of the dorsiflexors and plantar flexors, respectively, and the myotonometric properties (tone, stiffness, and elasticity) of the ankle periarticular muscles that perform the same motion as the tested muscle force (anterior tibialis, and separately for the medial and lateral gastrocnemius). The correlations were analyzed for both the injured and healthy lower limbs.

We found direct correlations between stiffness (assessed with MyotonPro) and isometric muscle force on both the affected and non-affected sides. The study by Chen S et al. [33] on recreationally trained male runners (age 20–23 years) found that ankle plantar flexor concentric and eccentric peak torque, and dorsiflexor eccentric peak torque at 60°·s^−1^ were significantly correlated with leg stiffness (the ratio between the maximal vertical ground reaction force and the maximum compression in the lower-limb length).

One of our previous studies evaluated the intra-rater reliability of the myotonometer and of the handheld MicroFET2 dynamometer in healthy controls after a 4-week interval between the first and second assessments. Frequency showed good to excellent reliability for all four assessed muscles (anterior tibialis, longus peroneus, medial gastrocnemius and lateral gastrocnemius). For stiffness the reliability was excellent (intra-class correlation coefficient more than 0.92 for all the assessed muscles). Decrement showed good to excellent the intra-rater reliability. Handheld dynamometry revealed excellent isometric muscle force measurement reliability of both ankle dorsiflexors and ankle plantar flexors (intra-class correlation coefficient 0.98 and 0.97, respectively) [9]. The assessments of the previous and current research were performed by the same investigator (A.-D.B.).

The current study of patients after a unilateral surgically treated ankle fracture (aged 30–59 years) reported inverse correlations between decrement and isometric muscle force on both the affected and non-affected sides. As the elasticity is inversely related to decrement, we noted a direct correlation between the elasticity of the anterior tibialis and the isometric force of the dorsiflexors of both injured and healthy lower limbs. The same findings were observed for medial and lateral gastrocnemius, respectively, and the isometric force of plantar flexors for both affected and non-affected sides. The study by Hasson et al. [34] assessed muscle series-elasticity measured during ramped dynamometer contractions using ultrasonography to quantify aponeurosis extension as a function of torque. Their results on 12 young (age range 21–31 years) and 12 older (age range 66–79 years) independent community-dwelling adults without musculoskeletal or neurological impairments demonstrated significant age-related modifications in ankle muscle dynamic and elastic properties.

The findings of our study have clinical significance for managing patients after a surgically treated tibial pilon fracture. After a successful surgical procedure, patients need further rehabilitation to regain ankle muscle strength on the injured side.

Previous research has shown that muscle stiffness is a valid, indirect measure of muscle activation and joint force production when measured with myotonometry [35,36].

Considering that, the use of both myotonometry and a hand-held dynamometry before the start of a physical exercise program will add important data on the periarticular ankle muscles of patients after an operated tibial pilon fracture. The two devices are easy to use, with assessments lasting approximately 15 min. Using these affordably priced tools during rehabilitation can help adapt the exercise program to each individual patient’s progress.

## 5. Limitations and Areas of Future Research

Our study’s cross-sectional design restricts our capacity to determine causal links between the variables studied. Although cross-sectional research offers useful insights into associations, longitudinal or interventional studies would better elucidate how muscle strength, biomechanics, and viscoelastic traits of ankle muscles interact over time in patients who have undergone tibial pilon surgery fractures. Our research will continue with patients who have completed a 3-month exercise program and will be assessed afterwards. The comparisons of myotonometric parameters and isometric muscle force before and after the 3-month exercise program will be the subject of a future paper. Moreover, we intend to compare the isometric muscle force, biomechanical, and viscoelastic properties of ankle periarticular muscles between patients who will follow a 3-month exercise program and healthy controls.

Our study sample may be biased toward individuals with right-sided tibial pilon fractures, potentially resulting in a higher proportion of right-sided participants. This could limit the extent to which our findings can be applied. Future research should focus on recruiting a more balanced group of participants to reduce this bias.

Additionally, a possible limitation is the presence of fixation hardware after ORIF. While myotonometric measurements were taken at standardized muscle belly sites, likely minimizing direct mechanical interference from implants, we cannot entirely rule out indirect effects. Factors such as postoperative scar tissue formation, changes in fascial mobility, soft-tissue remodeling, and variations in local loading conditions might affect the mechanical properties of the muscles being measured.

Another limitation is the interval between surgery and inclusion in the current study (12 to 24 months after surgery). Since pilon fractures represent less than 10% of all tibial fractures, we have extended the follow-up interval [37].

## 6. Conclusions

Myotonometry revealed heightened stiffness and mechanical stress relaxation time with concurrent lowered tone and elasticity of the anterior tibialis, longus peroneus, medial and lateral gastrocnemius muscles of the side with the operated tibial pilon fracture in comparison to the non-affected side. Tone, stiffness, and elasticity of the anterior tibialis and medial and lateral gastrocnemius were directly correlated with the isometric muscle force of dorsiflexors and plantar flexors, respectively.

## Figures and Tables

**Table 1:** Patient demographics.

Number of patients	39
Age (years)	42.3 (9.8)
Gender	
Men, *n* (%)	27 (69.2%)
Women, *n* (%)	12 (30.8%)
Height (cm)	176.2 (8.2)
Weight (kg)	93.9 (22.1)
BMI (kg/m^2^)	29.9 (5.85)

Data are shown as mean (standard deviation); *n*: number of patients; BMI: body mass index.

**Table 2:** Comparison of ankle range of motion between the affected and non-affected sides.

Motion	Affected Side	Non-Affected Side	*p*-Value
Dorsi flexion (°)	7.6 (2.4)	19.6 (5.7)	**<0.0001**
Plantar flexion (°)	26.5 (14.6)	39.6 (5.4)	**<0.0001**
Inversion (°)	19.2 (10.2)	26.9 (7)	**0.0003**
Eversion (°)	11.5 (8.5)	21.1 (8.4)	**<0.0001**

Data are shown as mean (standard deviation). Bold values: *p* < 0.05 indicates statistical significance.

**Table 3:** Comparison of the myotonometric parameters between the affected and non-affected sides.

Tested Muscle	Myotonometric Parameters	Affected Side	Non-Affected Side	*p*-Value
Anterior tibialis	Tone (Hz)	19.22 (4.58)	19.78 (4.86)	0.606
	Stiffness (N/m)	430.3 (148.4)	414 (149.1)	0.629
	Decrement	0.96 (0.16)	0.95 (0.15)	0.772
	Stress relaxation time (ms)	14.55 (4.07)	14.05 (3.82)	0.578
	Creep	0.92 (0.22)	0.89 (0.2)	0.458
Longus peroneus	Tone (Hz)	18.58 (4.3)	18.85 (4.27)	0.782
	Stiffness (N/m)	422.8 (135.7)	408.2 (121.9)	0.62
	Decrement	1.01 (0.2)	0.9 (0.18)	**0.021**
	Stress relaxation time (ms)	13.92 (3.73)	14.11 (3.7)	0.82
	Creep	0.89 (0.19)	0.88 (0.2)	0.933
Medial gastrocnemius	Tone (Hz)	14.7 (3.36)	15.42 (3.12)	0.329
	Stiffness (N/m)	317.6 (88.07)	277.2 (81.25)	**0.038**
	Decrement	1.24 (0.24)	1.19 (0.16)	0.251
	Stress relaxation time (ms)	20.16 (5.35)	18.69 (5.07)	0.217
	Creep	1.24 (0.32)	1.16 (0.3)	0.23
Lateral gastrocnemius	Tone (Hz)	16.19 (3.5)	17.28 (4.12)	0.21
	Stiffness (N/m)	368 (115.6)	322.2 (79.39)	**0.045**
	Decrement	1.34 (0.27)	1.33 (0.32)	0.84
	Stress relaxation time (ms)	17.84 (4.95)	16.49 (5.15)	0.243
	Creep	1.12 (0.3)	1.04 (0.31)	0.257

Data are shown as mean (SD). Bold values: *p* < 0.05 indicates statistical significance.

**Table 4:** Comparison of isometric muscle force between the affected and non-affected sides.

Isometric Muscle Force	Affected Side	Non-Affected Side	*p*-Value
Dorsiflexors (N)	130.2 (32.08)	161.9 (39.47)	**0.0002**
Plantar flexors (N)	163 (41.67)	190.1 (44.1)	**0.0066**

Data are shown as mean (standard deviation); N: Newton. Bold values: *p* < 0.05 indicates statistical significance.

**Table 5:** Correlations between muscle properties and isometric muscle force.

Muscle Properties	Affected Side		Non-Affected Side	
	Isometric Muscle Force of Dorsiflexors	Isometric Muscle Force of Plantar Flexors	Isometric Muscle Force of Dorsiflexors	Isometric Muscle Force of Plantar Flexors
Anterior tibialis: tone	r = 0.457 *p* = **0.003**	-	r = 0.76 *p* < **0.0001**	-
Anterior tibialis: stiffness	r = 0.577 *p* = **0.0001**	-	r = 0.735 *p* < **0.0001**	-
Anterior tibialis: decrement	r = −0.103 *p* = 0.531	-	r = −0.27 *p* = 0.096	-
Medial gastrocnemius: tone	-	r = 0.228 *p* = 0.151	-	r = 0.483 *p* = **0.0018**
Medial gastrocnemius: stiffness	-	r = 0.152 *p* = 0.151	-	r = 0.463 *p* = **0.003**
Medial gastrocnemius: decrement	-	r = −0.236 *p* = 0.147	-	r = −0.234 *p* = 0.15
Lateral gastrocnemius: tone	-	r = 0.228 *p* = 0.352	-	r = 0.268 *p* = 0.097
Lateral gastrocnemius: stiffness	-	r = 0.025 *p* = 0.87	-	r = 0.406 *p* = **0.01**
Lateral gastrocnemius: decrement	-	r = −0.378 *p* = **0.017**	-	r = −0.469 *p* = **0.002**

*p*: *p*-value; r: rank correlation coefficient. Bold values: *p* < 0.05 indicates statistical significance.

## Data Availability

The data presented in this study are available upon request from the corresponding author (G.-B.H.).

## Abbreviations

The following abbreviations are used in this manuscript:

ORIF	Open Reduction and Internal Fixation
SD	Standard Deviation
